# Changes of in vivo electrical conductivity in the brain and torso related to age, fat fraction and sex using MRI

**DOI:** 10.1038/s41598-024-67014-9

**Published:** 2024-07-12

**Authors:** Zhongzheng He, Paul Soullié, Pauline Lefebvre, Khalid Ambarki, Jacques Felblinger, Freddy Odille

**Affiliations:** 1 IADI U1254, INSERM and Université de Lorraine, Nancy, France; 2 Siemens Healthcare SAS, Saint Denis, France; 3 CIC-IT 1433, INSERM, Université de Lorraine and CHRU Nancy, Nancy, France

**Keywords:** Predictive markers, Magnetic resonance imaging, Biological physics

## Abstract

This work was inspired by the observation that a majority of MR-electrical properties tomography studies are based on direct comparisons with ex vivo measurements carried out on post-mortem samples in the 90’s. As a result, the in vivo conductivity values obtained from MRI in the megahertz range in different types of tissues (brain, liver, tumors, muscles, etc.) found in the literature may not correspond to their ex vivo equivalent, which still serves as a reference for electromagnetic modelling. This study aims to pave the way for improving current databases since the definition of personalized electromagnetic models (e.g. for Specific Absorption Rate estimation) would benefit from better estimation. Seventeen healthy volunteers underwent MRI of both brain and thorax/abdomen using a three-dimensional ultrashort echo-time (UTE) sequence. We estimated conductivity (S/m) in several classes of macroscopic tissue using a customized reconstruction method from complex UTE images, and give general statistics for each of these regions (mean-median-standard deviation). These values are used to find possible correlations with biological parameters such as age, sex, body mass index and/or fat volume fraction, using linear regression analysis. In short, the collected in vivo values show significant deviations from the ex vivo values in conventional databases, and we show significant relationships with the latter parameters in certain organs for the first time, e.g. a decrease in brain conductivity with age.

## Introduction

Electrical properties (EPs), namely conductivity $\upsigma$ and permittivity $\upepsilon$, are fundamental properties of matter that quantify the degree and nature of its interaction with electromagnetic fields. In human tissues, they are closely related to important biological quantities such as ionic concentration, intra- or extracellular volumes and local architectures. As dispersive parameters, they also vary with the operating frequency, suggesting the possibility of multispectral tissue characterization^1^. These reasons suggest that they would make useful new biomarkers for the clinical use and that they would provide a better understanding of the interactions between electromagnetic waves and the human body^2^. Obtaining reliable in vivo EP values, and knowing their variability in the population, is especially important when considering the use of standard electromagnetic models for Specific Absorption Rate (SAR) estimation^3^. Better modelling might reduce safety margins and, ultimately, examination times. At the same time, the evolution of conductivity in relation to patient characteristics may provide new clues for understanding certain physiological mechanisms, such as aging.

Historically, classical methods for estimating EPs in tissues were performed on excised pieces of tissue^4,5^. In recent decades, however, theoretical and technical efforts have enabled in vivo measurements, thanks to the combination of stimulation hardware and numerical capabilities for solving challenging problems. As a result, invasive current-injection imaging techniques^6^ now coexist with induction-based methods^7^ over a wide frequency range. From this point of view, it was soon realized that MRI, beyond its imaging capabilities, could also be used as a non-invasive induction setup^8,9^. In the specific MR radiofrequency range, this led to the development of a specific research field now known as MR-Electrical Properties Tomography (MR-EPT)^10,11^.

One of the main features of MRI is the manipulation of a radiofrequency (RF) field – the transmit field ${\text{B}}_{1}^{+}$ – at the Larmor frequency, to induce resonance. At the same time, these radio waves interact with tissues, forming currents whose imprint can be identified in the measured signal – the receive field ${\text{B}}_{1}^{-}$ – after reception. The aim of MR-EPT is to isolate this signature in the MR signal and infer tissues EPs from an approximate mathematical model. Beyond safety issues, best documented for MRI^12^ than for current-injection procedures^13^, the main advantage of MR-EPT is that it can be easily integrated into a standard MRI examination. In addition, it naturally takes advantage of the high-resolution capacity of MR images to better constrain the solving of the inverse problem at hand.

In its simplest form, it is formulated as a homogeneous Helmholtz equation involving one or both RF components of the signal^10,14–16^, ${\text{B}}_{1}^{+}$ and ${\text{B}}_{1}^{-}$ where, more specifically, tissue EPs are derived directly from the spatial second derivatives of the fields. Although very straightforward, this procedure lacks precision as the calculation of second derivatives is very sensitive to the inherent noise of MRI^17,18^, and because it makes strong assumptions on both the signal and Maxwell equations for sake of simplification^19–21^. In particular, it assumes that spatial variations of EPs are piecewise constant. A common strategy to mitigate noise amplification is to use large voxel sizes, specific differentiation kernels or strong image filters^18,22–26^ at the cost of amplifying edge effects at tissue boundaries. Meanwhile, numerous attempts have been made to improve the quality of the maps reconstructed in MR-EPT. A first class of advanced methods strives to maintain a direct equation model, closer to physical reality, i.e. not assuming piecewise constant EPs, at the cost of greater sensitivity to noise^27–32^. Another class of advanced methods, which have yet to be validated for in vivo use, employ more general models that may be more faithful to the original Maxwell equations, however they result in a more complex signal equation^33–36^, sometimes involving non-measurable quantities, such as the scattered RF electrical field. The last class of advanced methods can be termed data-driven, including methods based on supervised machine learning^37–40^ and those using models for signal matching^41,42^. Importantly, the latter methods make use of a priori information on the data (explicitly or implicitly) or the result to be obtained. While a prior is often needed to better constrain the problem, it can also introduce an undesired bias preventing absolute quantification, e.g. if the solver was trained to match the ex vivo values.

Many studies have also attempted to provide reference in vivo EP values, associated with specific organs or pathologies such as brain tissues^16,42–44^, brain tumors^45,46^, breast lesions^47–49^, musculoskeletal tissues^50^, pelvis^51^ or rat brain^52^ and liver^53^. These works have focused on conductivity rather than permittivity, because permittivity reconstruction is even more prone to noise^54^. Given the inherent sensitivity and variability of MR-EPT techniques, large discrepancies in conductivity values are found although the orders of magnitude are close. Interestingly, the simplest formulation of MR-EPT has been used in such studies, both because it is very convenient and because advanced methods are difficult to adapt to in vivo data. Even though voxel-wise estimation is still very challenging with such methods, focusing on an average value for the organ of interest greatly tempers the effect of noise^37^. Authors have compared the measured in vivo values to ex vivo databases, but such comparison should be done carefully since the conditions are very different. In short, existing in vivo studies show significant deviations from these ex vivo reference values, when these ex vivo values are not used as a prior for the reconstruction^43,55,56^. A recent review of the literature on variations in brain tissue conductivity highlights the uncertainties in this regard, emphasizing that correlations with individual conditions – such as pathologies – are similar however ^57^.

In this study, we therefore sought to estimate macroscopic values of conductivity in several organs (brain and torso) in a small adult population (17 volunteers) from complex images of a 3D UTE sequence, and using a homogeneous Helmholtz MR-EPT formulation^15,19,32,58–60^. We then explored the relationship of these quantities with biological parameters such as age, Fat Volume Fraction (FVF), BMI (Body Mass Index) or sex, and identify relevant correlations.

## Materials and methods

This study was approved by an ethics committee (Comité de Protection des Personnes Sud Est III) and informed written consent was obtained (ClinicalTrials.gov identifier: NCT04645628, first trial registration 27/11/2020). The study was supported by CIC-IT Nancy, sponsored by CHRU Nancy (Département Méthodologie, Promotion, Investigation), and carried out in accordance with the relevant guidelines and regulations.

### Simulation

The proof of concept of this study was performed using a simple EM simulation with the commercial software CST Studio Suite 2023 (V6R2023x.FP.CFA.2419, https://software.3ds.com, Computer Simulation Technology, Dassault Systemes, France). A two-port quadrature birdcage coil and a multi-compartment cylindrical conductive volume wereq modeled and used to simulate both RF transmit and receive fields at 123 MHz. The outer diameter of the cylindrical volume is 30 cm, comparable to the dimensions of a human body, and the concentric sections are between 3 and 4 cm. The conductivity range has been chosen to be representative of most tissues. The composite RF field data were then exported to MATLAB, where we added artificial Gaussian noise to obtain an SNR comparable to our experimental images. Reconstructed conductivity values were compared with the reference values in both noiseless and noisy models.

### Study population

The average conductivity standard deviation of previous studies^16,55^ can be approximated at 0.3 S/m for the best results.The sample size required to provide a 95% confidence interval with a 0.16 S/m margin of error is fourteen^50^. Assuming a dropout rate of 15%, the MR-EPT study was performed on seventeen volunteers (10 males & 7 females) aged between 25 and 73 years old (mean = 43.5, SD = 16.9), with body mass index range from 19.6 to 32.6 kg/m^2^ (mean = 24.5, SD = 2.6). Besides, this number of volunteers allows us to test a maximum of two predictors.

### MRI measurements

Imaging studies were performed on a 3 T MAGNETOM Prisma MRI scanner (Siemens Healthineers, Forchheim, Germany), with standard quadrature birdcage coil for transmission. A 20-channel and 18 + 20-channel phased array coils were respectively used in receive mode for brain and torso mapping.

For the brain study, a reference anatomical volume was acquired using a conventional MPRAGE sequence while complex images from a UTE SpiralVIBE prototype^61,62^ were used for assessing conductivity. It consists in a stack-of-spirals trajectory with adaptive TE and variable-duration slice encoding to minimize ${\text{T}}_{2}^{*}$ decay^63^. The relevance of this type of sequence has been demonstrated in the literature for its significant RF weighting^15,60^ and robustness to off-resonance effect and eddy currents.

Regarding torso imaging, UTE images were acquired during a breath-hold period, optimizing resolution, k-space acquisition scheme and slice direction. Reference Water/Fat images were obtained using a standard VIBE DIXON sequence in coronal view. FVF maps were generated from the ratio F/(W + F) applied to each organ. We should point out that we used a dual-echo acquisition to reduce breath-hold time, and this could also lead to a bias in the exact estimation of fat fraction^64^. If this bias is consistent across all organs, however, the trends measured should be similar.

In each case, an optimal 2-port transmission technique was used^65^, and receive coil combination was achieved for UTE imaging using the vendor’s reference-based adaptive approach^66^ which mimics the data from a complete body coil scan. Phase unwrapping was applied when needed as a simple constant shift since the range of phase variations for our applications lies into [$-\uppi ,\uppi$]. Moreover, an acceleration factor of 2 was also used with the SPIRiT ^67^ method to reduce acquisition time, without compromising the reconstruction accuracy^44^.

For both tissue classes, the specific sequence parameters are reported in Table 1.

**Table 1:** MR sequences parameters.

Sequence	Brain		Torso	
	MPRAGE	UTE SpiralVIBE	VIBE DIXON	UTE SpiralVIBE
TE (ms)	2.45	0.05	1.03–2.13	0.05
TR (ms)	2300	7.56	4.06	2.73
TI (ms)	900	/	/	/
Flip Angle (°)	9	3	11	3
FoV (mm)	270	270	479	500
Reconstruction Matrix	256 × 256 × 176	224 × 224 × 160	224 × 224 × 110	256 × 256 × 96
Bandwidth (Hz/pixel)	250	2232	1063	2170
Pixel Spacing (mm)	1.06	1.21	2.14	1.95
Slice Thickness (mm)	1	1.2	2	2.5
Echo Train Length	1	3	2	1
Parallel Acceleration Factor	3 (in-plane)	2	3 (out-of-plane)	2 (out-of-plane)
Acquisition Time	4 min 9 s	9 min 1 s	16.8 s	17.3 s

TE, Echo time; TR, Repetition time; TI, Inversion time; FoV, Field-of-view. Note that Dixon sequence needs two TE for water and fat imaging.

### Registration and segmentation

**Figure 1:**
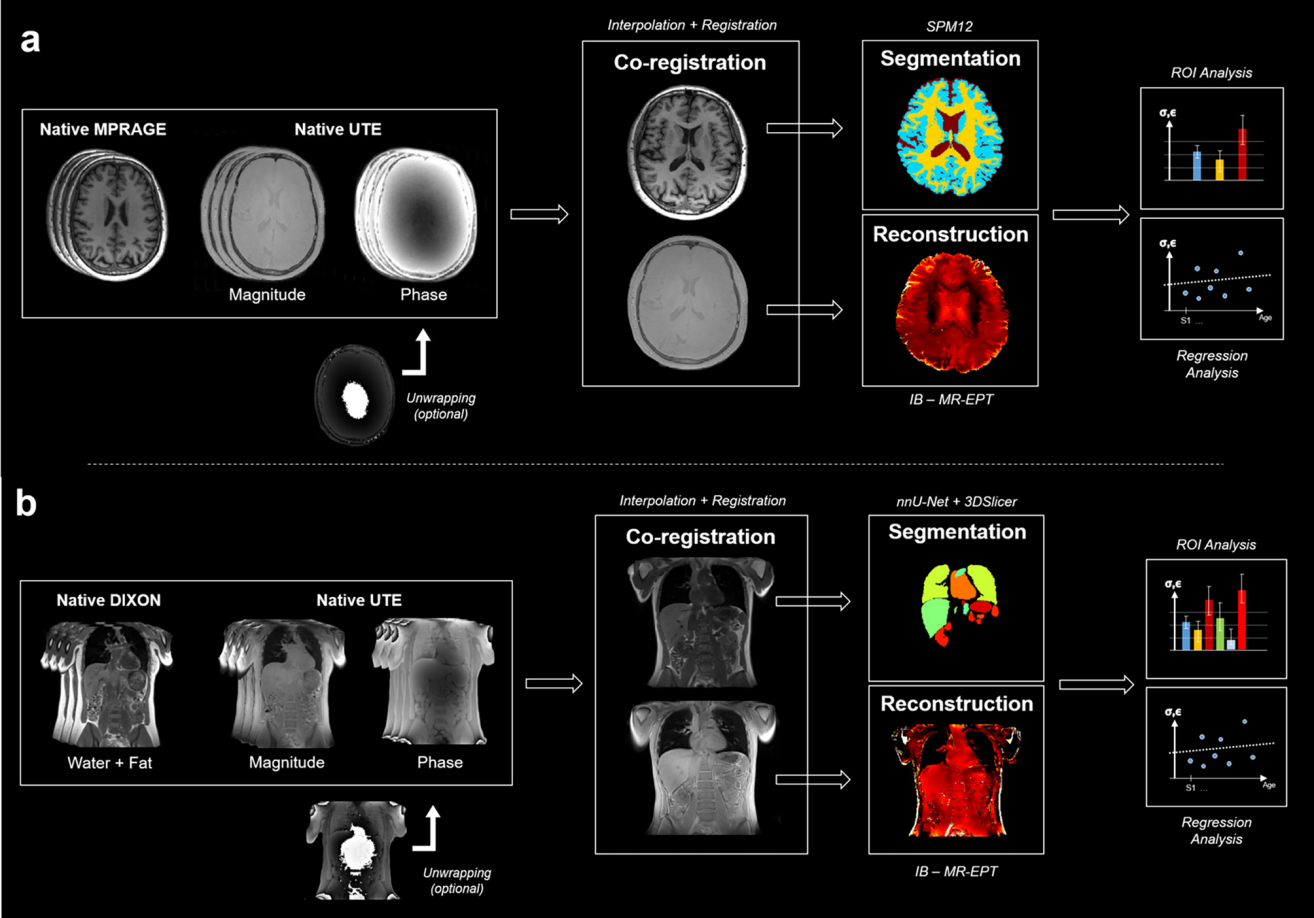
Representation of the reconstruction pipeline for brain (**a**) and torso (**b**) images. Native MPRAGE or VIBE DIXON images are first re-aligned to their respective UTE volumes, then segmented using SPM12 or a combination of nnU-Net and manual delineation.The registered segmentation masks provide both a contrast prior (for constrained estimation of the Laplacian), and the ROIs for subsequent statistical analysis.

Firstly, in each case, the reference images (i.e., MPRAGE and VIBE DIXON images) were resampled and re-aligned to the UTE magnitude images using spline interpolation and a total-variation-regularized non-rigid registration algorithm^68^, respectively (Fig. 1). Then for organ-specific region-of-interest (ROI) analysis, we used SPM's segmentation tool (SPM12, London, UK) to post-process the brain MPRAGE registered volume to segment gray matter (GM), white matter (WM) and cerebrospinal fluid (CSF). For torso organ segmentation, we used a semantic nnU-Net pipeline^69^ applied to the AMOS22^70^ dataset. This specific database contains images labelled with fifteen different tissue classes and was the basis of the recent Multi-Modality Abdominal Multi-Organ Segmentation Challenge (MICCAI 2022). The network was implemented with PyTorch^71^ and trained in about 8 h on a GPU (NVIDIA Quadro RTX 5000). After training, we applied it to our registered VIBE DIXON data and segmentations took about one minute per 3D volume. In addition to the tissues labeled in the AMOS database, visceral abdominal fat (VAT), heart and lungs were added semi-automatically from 3DSlicer^72^.

### Conductivity reconstruction

**Figure 2:**
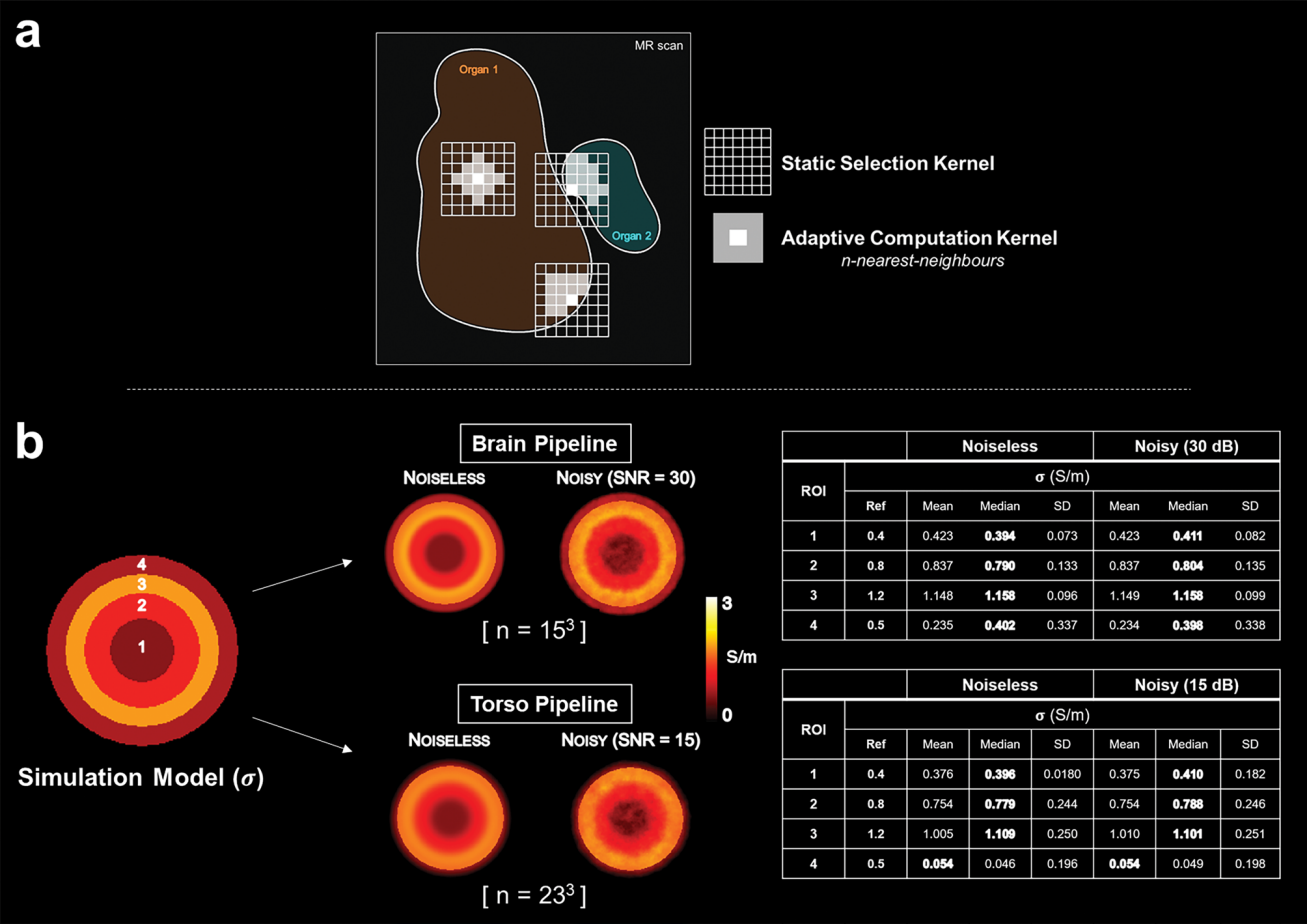
Simulation study as proof of concept. The bi-adaptive kernel (**a**) optimizes the fitting area and constrains the homogeneity of the Laplacian estimate, based on both contrast proximity and a fixed number of voxels. Both reconstruction pipelines are simulated (**b**) and the reconstruction results compared with the conductivity model in each case, with and without noise. The respective SNRs are defined according to the values obtained in our in vivo images. The associated tables show the results for each ROI, noting the closest proximity of the median to the reference values.

After acquisition, the images were transferred to a workstation and processed using MATLAB (The MathWorks, Natick, MA, USA). The choice of a UTE sequence makes it possible to reconstruct conductivity maps directly from complex images, i.e. the combination of magnitude and phase, according to the following relationship ^15,19^:

$$ \sigma = {\text{Re}}\left( \kappa \right) = {\text{Re}}\left( {\frac{1}{{{\text{i}}\mu \omega }}\frac{{\Delta \sqrt {{\text{B}}_{1}^{ + } {\text{B}}_{1}^{ - } } }}{{\sqrt {{\text{B}}_{1}^{ + } {\text{B}}_{1}^{ - } } }}} \right) \approx {\text{Re}}\left( {\frac{1}{{{\text{i}}\mu \omega }}\frac{{\Delta \sqrt {{\text{S}}_{{{\text{UTE}}}} } }}{{\sqrt {{\text{S}}_{{{\text{UTE}}}} } }}} \right) $$

where $\kappa \underline{\underline{{{\text{def}}}}} \sigma + {\text{i}}\omega \in$ is known as complex admittivity, $\upmu$ is the local magnetic permeability, assumed to be equal to that of the vacuum ${\upmu }_{0}$ in the RF range, $\omega =\gamma {\text{B}}_{0}$ is the Larmor frequency, $\Delta$ is the Laplacian operator and ${\text{B}}_{1}^{+}{\text{B}}_{1}^{-}$ is the RF fields product. From a theoretical point of view, this relationship is obtained from a combination of two homogeneous Helmholtz equations applied to ${\text{B}}_{1}^{+}$ and ${\text{B}}_{1}^{-}$, multiplied by ${\text{B}}_{1}^{-}$ and ${\text{B}}_{1}^{+}$ respectively, to yield the Laplacian of the product ${\text{B}}_{1}^{+}{\text{B}}_{1}^{-}$. The square root appears after the neglected terms have been omitted. The magnitude and phase of $\sqrt{{\text{B}}_{1}^{+}{\text{B}}_{1}^{-}}$ are defined straightforwardly as the magnitude of the product root and half of its phase, assuming proper unwrapping beforehand. Suitability of the UTE sequence here depends on the use of a small flip angle, allowing the product ${\text{B}}_{1}^{+}{\text{B}}_{1}^{-}$ to appear directly in the signal^73^ as ${\text{S}}_{\text{UTE}}={\text{I}}_{0}{\text{B}}_{1}^{+}{\text{B}}_{1}^{-}$ (${\text{I}}_{0}$ = density and relaxation contrasts). This formulation is more general than those involving only the RF phase component^30^ and more accurate when the correct assumptions are satisfied^15^, essentially that EPs and ${\text{I}}_{0}$ contrasts are piecewise constant i.e. $\nabla\upkappa =0$ and $\nabla {\text{I}}_{0}=0$. In this study, the Laplacian was calculated for each target voxel using a bi-adaptive strategy with a constrained Savitzky-Golay kernel. First, a large selection kernel size^31^ was used to identify voxels with signal intensity close to the central target voxel. This was done by selecting voxels that belonged to the same segmentation class as the central target voxel, using the segmentation masks obtained from the reference MPRAGE/DIXON images. Once this first mask was obtained, we kept the n nearest neighbors in the sense of the Euclidean distance^74^ for the actual fitting process. This bi-adaptive strategy allowed to: (i) limit the bias at the boundaries between tissues; (ii) keep the number of voxels sufficiently large for the Savitzky-Golay fit, thereby preventing noise amplification (Fig. 2). The size of the calculation kernel was adapted for each component of this study (brain: N = 15^3^ and torso: N = 23^3^) as the image resolutions were slightly different. This appeared to be the best compromise for preserving final resolution (brain: 1.2 × 1.2x1.2 mm^3^, torso: 1.95 × 1.95x2.5 mm^3^) and mitigating the effect of noise. Note that no pre- or post-filtering was used to avoid undesired smoothing effects. Conductivity values were finally estimated within the organ-specific ROIs as mean, median and standard deviation. These ROIs are built from individual segmentations where each organ is uniquely labeled, and on which we perform a single-voxel erosion to reduce edge effects.

### Statistical analysis

For this analysis, we included the three types of brain tissue (WM/GM/CSF), as well as fourteen classes of torso tissues from the segmentation step: [Spleen, Right and Left Kidneys, Gallbladder, Esophagus, Liver, Stomach, Aorta, Postcava, Pancreas, Duodenum] from nnU-Net^69^ and [Heart, Lungs, Visceral Fat (VAT)] from manual segmentation.

Data were pooled and grouped according to tissue types, in order to determine (i) an estimate of mean/median/SD conductivity for each tissue, (ii) which significant independent variables account for differences in conductivity, and (iii) any extended relationship between conductivity and reported significant variables.

Boxplot diagrams, presenting the ROIs medians, means and standard deviations of conductivity measurements in our cohort were generated to display variations in conductivity within different tissue classes. We also provide metrics on the number of voxels associated with each ROI, as well as Fat Volume Fraction (FVF) statistics for torso organs only. Note that these numbers of voxels are an important reliability criterion: the higher they are, the more effective the compensation for reconstruction errors.

We aimed to test the independent influence of sex, age, BMI or FVF on tissues conductivity (Two-tailed Pearson’s correlation) with a linear model using the MATLAB fit linear model function. Pearson correlation coefficient r and p values for all estimators were given, and p values below 0.05 were considered to indicate statistical significance. Once the univariate regressions have been obtained, and if two predictors are significant, we use multivariate models (glmfit) to test for weighted dependence. The correlation between predictors is also estimated using regression. Linear regression curves were generated for univariate models.

## Results

Brain data from all volunteers (n = 17) were used for the analyses whereas only fifteen torso data were usable. One of the volunteers had a known intestinal pathology, and an incidental finding was made in another one. They were excluded from the subsequent statistical analyses, but we do provide reconstruction data for illustrative purposes in the Supplementary Material (Fig. S1).

### Simulation study

The use of a bi-adaptive computation kernel optimizes the role of the prior in the reconstruction process as it allows a voxel selection that matches the piecewise constant model equation. A simple illustration is provided in Fig. 2. As the typical SNR of our brain images was ~ 30 dB, and that of our torso images was ~ 15 dB, we mimicked both reconstruction pipelines and adapted the size of the computation kernel accordingly. While it maintains a very good accuracy in each ROI without excessive smoothing, it is also robust to noise (Fig. 2). There are however some visible deviations due to the lack of signal at the edge of the calculation area, as well as visible inconsistencies due to inherent noise amplification. SD values reflect both the effect of smoothing and noise. It is also worth noting here that the median is a better absolute estimator than the mean for central compartments.

### Conductivity maps

**Figure 3:**
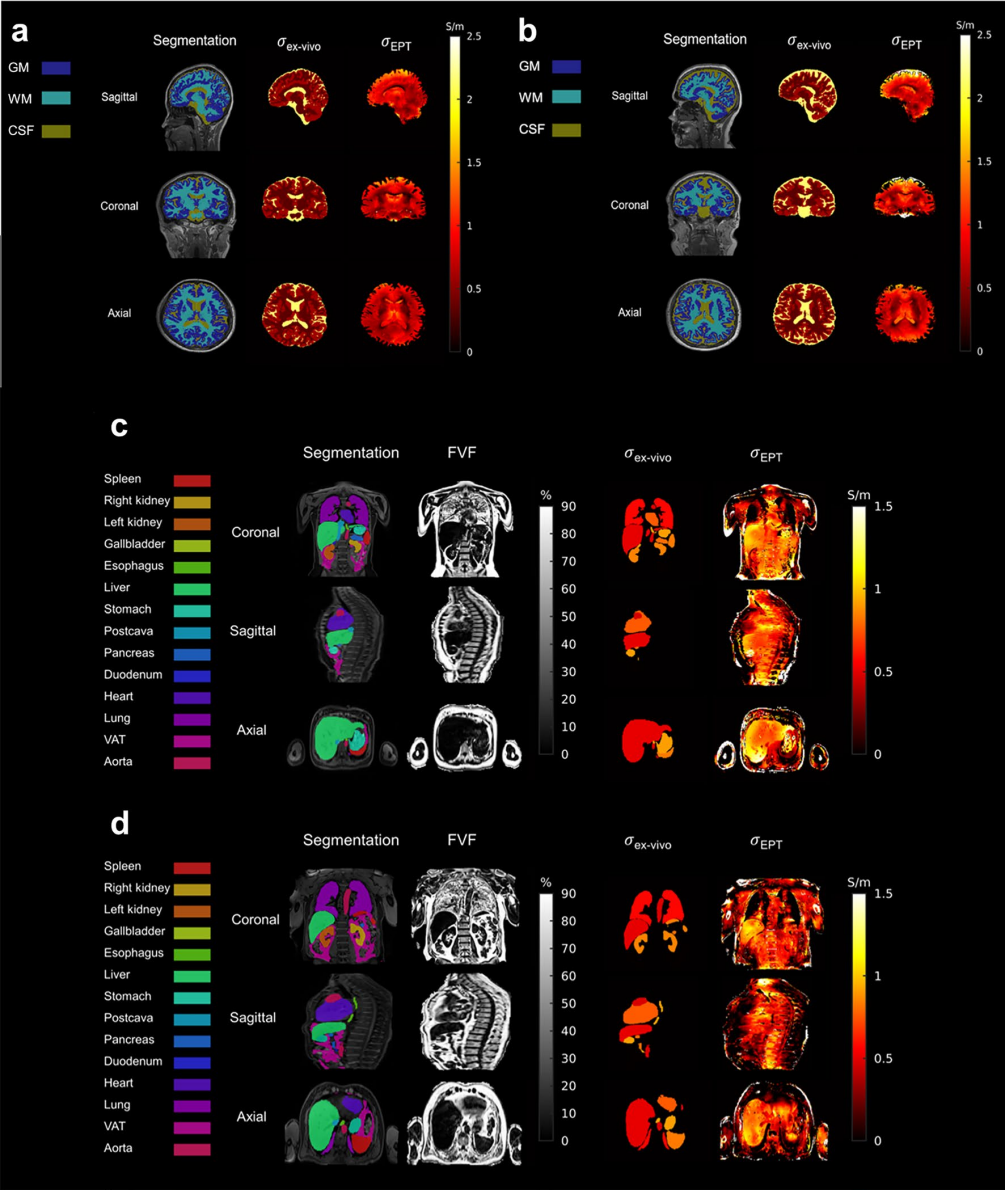
Reconstruction of brain conductivity for two different volunteers (**a**, **b**) with their correspondent segmentation and reference maps. Note here the lack of conductivity matching in the ventricles (CSF) due to the insufficient signal in the UTE magnitude map. Similarly, torso conductivity maps (**c**, **d**) for another two volunteers, segmentation and reference maps, as well as estimates of fat fraction. Boundary artefacts at the periphery of the two volumes are clearly visible.

Examples of reconstructed maps are shown in Fig. 3 with associated ROIs. The visible segmentations were subsequently eroded for statistical analysis, as the decrease in quality of conductivity maps at the periphery or in certain transition zones, such as the CSF or the heart, quickly became apparent. We also note that the proposed technique resulted in non-physical (negative) conductivity values in the lung. Reconstructed images of subjects with known or incidentally discovered conditions are shown in the supplementary information for information purposes only (Fig. S1).

### Population values

**Figure 4:**
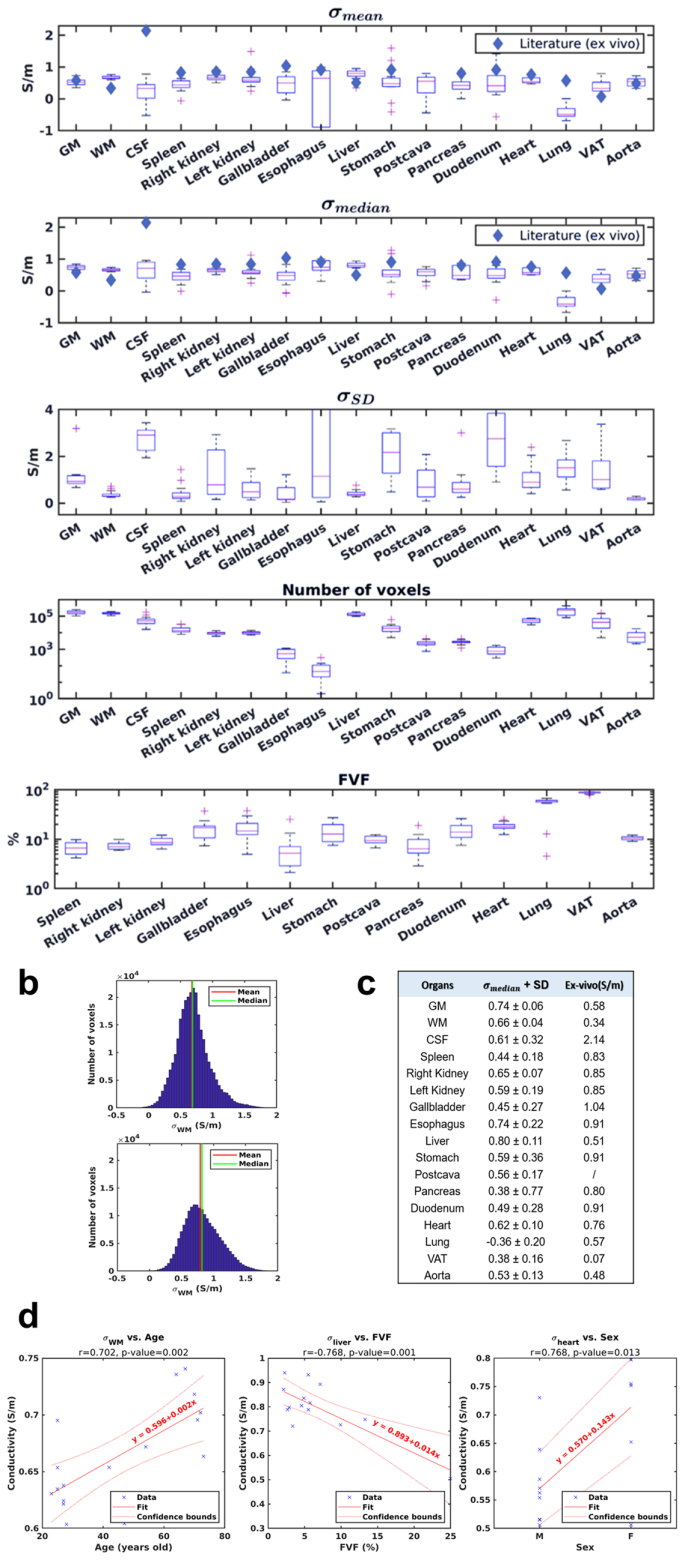
Population statistics. Boxplot representations (**a**) of the population distribution of mean conductivity in each organ, median conductivity, standard deviation as well as the associated mean number of voxels and volume fat fraction. Colored diamonds represent ex vivo reference conductivity values. Example histograms (**b**) for a given ROI (WM) in two different volunteers show limited deviation from a Gaussian distribution. Summary table (**c**) of the population average of the median conductivity values in each organ compared with the corresponding reference value on which linear regressions are performed (**d**). The regression equations are shown in red and 95% confidence bounds are depicted in dotted lines. Each prediction variable is tested for each organ in a similar way.

Means, medians, and SD of conductivity for all selected organs are shown in Fig. 4a. We also provide reference conductivity values from the ex vivo literature for comparison when available. The obtained EP values are close yet not equal to the reported literature values. Significant deviations are observed in some ROIs but no obvious pattern seems to emerge, in particular we do not observe a consistent bias between in vivo and ex vivo values. The example of a histogram associated with the estimation of conductivity in the WM in two different subjects is also shown (Fig. 4b), which suggests the near-normality of the data, and the validity of the analysis in terms of mean/median. As suggested earlier, we noticed that the median tends to be more stable over organs, likely due to the weight of certain outliers in the calculation of the means. Therefore the median values were used for the statistical analyses (Fig. 4c). A focus on standard deviations and the average number of voxels helps identify the most reliable regions (e.g. GM, WM, Spleen, Liver, and Pancreas).

Excluding outliers, the ranges of ROIs median conductivities were as follows (min–max): Grey Matter, 0.65–0.85 S/m; White Matter, 0.60–0.74 S/m; Cerebrospinal Fluid, -0.03–0.96 S/m; Spleen, 0.19–0.68 S/m; Right Kidney, 0.51–0.74 S/m; Left Kidney, 0.40–0.68 S/m; Gallbladder, 0.20–0.84 S/m; Esophagus, 0.30–1.10 S/m; Liver, 0.50–0.94 S/m; Stomach, 0.27–0.97 S/m; Postcava, 0.29–0.76 S/m; Pancreas, 0.35–0.87 S/m; Duodenum, 0.28–0.80 S/m; Heart, 0.51–0.80 S/m; Lung, -0.67–0.00 S/m, Visceral Adipose Tissue (VAT), 0.10–0.67 S/m; Aorta, 0.33–0.72 S/m. Negative values reflect the limits of the technique when SNR is very low, as in the lungs, or at the edge of the reconstruction domain, as in the CSF.

### Regressions and correlations

Univariate linear regression models allow a quick account of general trends, as shown in Fig. 4d. We observe, for example, that conductivity seems to evolve positively with age in the white matter (*p* = 0.002), negatively with FVF in the liver (*p* = 0.001, r = 0.70), and positively in the heart (*p* = 0.013, r = 0.77) for men compared to women (M to F, r = 0.77). All these trends are summarized in Table 2, with the corresponding p-value indicated. Significant correlations are shown in bold and the curves associated are provided as additional information (Fig. S2). Results for multivariate models are presented as additional information when more than one predictor was significant (Table S1). These results must also be balanced with the trust placed in population values. FVF values in the lung were excluded due to the SNR of the DIXON images being nearly zero. Interestingly, conductivity of the three major brain areas is positively and significantly correlated with age, while that of other organs is always negatively correlated. For seven of them, the link is significant or close to it; a larger cohort would allow these findings to be refined. We also note a discrepancy between the two kidneys, which could indirectly reflect a functional asymmetry in our population. FVF seems to be a particularly good predictor of liver and spleen conductivity unlike BMI, which relates only to the duodenum. Finally, sex seems to have a discriminating effect on certain organs, notably the liver and pancreas.

**Table 2:** Statistical results for monovariate regressions.

Organs	Age		FVF		BMI		Sex (M to F)		Predictors correlation
	r	*p*-value	r	*p*-value	r	*p*-value	r	*p*-value	*p*-value
**GM**	**0.506**	**0.038**			0.189	0.467	0.244	0.345	
**WM**	**0.702**	**0.002**			0.257	0.320	− 0.164	0.528	
**CSF**	**0.559**	**0.002**			0.391	0.702	− 0.624	0.542	
**Spleen**	− 0.517	0.059	− **0.719**	**0.004**	− 0.518	0.058	0.335	0.242	
**Right Kidney**	− **0.697**	**0.006**	− **0.601**	**0.023**	− 0.362	0.203	0.177	0.545	0.640
**Left Kidney**	− 0.507	0.064	− 0.172	0.557	− 0.413	0.142	0.274	0.342	
**Gallbladder**	− 0.187	0.523	0.285	0.323	− 0.420	0.135	0.509	0.063	
**Esophagus**	− 0.213	0.465	− 0.391	0.167	0.027	0.927	0.517	0.058	
**Liver**	− 0.353	0.216	− **0.768**	**0.001**	− 0.405	0.151	**0.767**	**0.001**	0.603
**Stomach**	− 0.483	0.080	− 0.393	0.165	− 0.486	0.078	**0.670**	**0.009**	
**Postcava**	− 0.523	0.055	0.120	0.683	− 0.425	0.129	0.422	0.133	
**Pancreas**	− **0.545**	**0.044**	− 0.519	0.057	− 0.520	0.057	**0.613**	**0.020**	**0.003**
**Duodenum**	− 0.380	0.181	0.300	0.297	− **0.686**	**0.007**	**0.669**	**0.009**	0.954
**Heart**	− 0.250	0.389	− **0.639**	**0.014**	− 0.354	0.214	**0.644**	**0.013**	0.322
**Lung**	− 0.219	0.452	**N/A**	**N/A**	− 0.129	0.660	**0.832**	** < 0.001**	
**VAT**	− 0.245	0.399	− 0.442	0.113	− 0.317	0.269	0.472	0.088	
**Aorta**	− **0.651**	**0.012**	− 0.282	0.329	− 0.360	0.207	**0.705**	**0.005**	**0.003**

Four different predictors (Age, FVF, BMI, and Sex) are tested for each organ. p-values < 0.05 are shown in bold, as are the associated correlation coefficients. Correlations between predictors are shown to illustrate the importance of multivariate analysis when two explanatory variables are significant.

## Discussion

This study provides new insights for tissue conductivity characterization with MRI, by focusing on macroscopic measurements and their dependence on general individual factors. It shows that it is possible to identify trends by looking at the macroscopic conductivity of each organ. We chose the median as a simple preferred metric as it seemed more robust than the classical mean, but a next step might be to look closer at the histograms within each ROI^45^. The use of a single UTE sequence and the associated simple EPT formulation also makes post-processing easier, as well as its integration into a standard imaging protocol.

Regarding the reconstruction method used in this work^15,75^, the ZTE/UTE-based framework has the advantage of cleverly reducing the weight of certain assumptions made in the more conventional MR-EPT models. Firstly, it avoids the need for the so-called “transceive phase assumption” (TPA) as the total RF phase is used and not an estimate of the transmit phase which is not directly measurable^10,14^. In theory, this means that the method can be used with any combination of transmit/receive antennas, provided that the combination is optimal and the SNR is sufficient^15,76^. The TPA suffers especially from geometric asymmetries as it works best for cylindrically symmetrical setups, and becomes increasingly invalid as the tissue is further away from the magnet's center^16^. The use of formulations involving the product of RF fields, and not ${\text{B}}_{1}^{+}$ and/or phase alone, enables reconstructions that are more accurate because they better compensate for inhomogeneities between transmit and receive fields while reducing errors associated with model simplification in each case^15,19^. In this study, the pipeline applied to the brain fulfilled these geometric conditions better than for the torso, where estimation deteriorated more markedly in the peripheral regions. This is also why small or tortuous structures, such as the esophagus or duodenum for which the SD is very large, are less amenable to this kind of analysis. Finally, unlike computationally intensive reconstruction schemes^28,33,35,37^, the implementation of our algorithm is fairly light: the calculation time for a brain volume, for example, is of the order of 5 min.

In addition, a recent study showed that the selected MR-EPT formulation provides satisfactory conductivity values compared with an external absolute probe reference in large homogeneous regions^32^. Its in vivo use requires proper boundary error control, which remain a serious issue for clinical applications^18,21^. As for Laplacian estimation, the use of a second order Savitzky-Golay kernel has been shown theoretically to be the best linear method to avoid noise amplification^17^. By constraining it to an anatomical prior, we artificially recreate the homogeneity criterion that "forces" the conditions of application of the model^23,55^. The use of both a large selection window^31^ and a restricted calculation window with a constant number of voxels (brain: n = 15^3^; torso: n = 23^3^), further limits potential differences between central and peripheral voxels within the same ROI. Our simulation study shows that this procedure works as long as the conductivity model is piecewise constant and we might expect this not to be true of extended structures such as white matter or the liver. The piecewise-constant assumption is nevertheless a preliminary prerequisite for deriving consistent macroscopic values and taking a step towards standardization for more realistic EM models. The consistency of conductivity values in the WM across our population (0.66 + /- 0.04 S/m) is a valuable piece of evidence in the validation of in vivo conductivity measurements. One problem, however, lies in estimating and eliminating the contrast term ${I}_{0}$, which could temper the latter. Filtering strategies can be used at the cost of degraded resolution^19^.

To calculate the SAR accurately, the permittivity must also be known. However, it can be estimated with a slight underestimation using conductivity alone and a theoretical permittivity model^77^. From a practical point of view, and even if we have not studied permittivity in this work, our pipeline makes it possible to consider its simultaneous reconstruction, as well as that of complex admittivity, which could also be a useful EM biomarker^19^. Extending the database will reduce the uncertainty regarding these quantities.

In terms of estimated values, we first observe that we tend to overestimate conductivity values in GM and WM compared to previous studies^10,16,44,55,56^. While GM conductivity is still greater than WM conductivity, it is about 0.3 S/m greater than the expected values and the gap between the two tissue types is reduced^3^. These discrepancies could be explained by a systematic error propagated in our reconstruction pipeline : depending on whether the phase is used alone or not, many authors point to under- or over-estimates compared to reference values^10,50^. In our case, the overestimation, already found in in reconstructions from ZTE images^15^, could be related to Gibbs-ringing^51^. Future work will quantify this effect, by adjusting reconstruction matrix sizes. The low standard deviation for these two regions in our population nevertheless shows that these measurements are reliable and can be used to identify statistical trends. The values found for CSF, on the other hand, appear unreliable due to image edge effects and are probably subject to the pulsatile effect described previously^78^. Regarding values for the torso regions, we also note an overestimation of 0.3 S/m compared with previous published results for the largest ROIs, i.e. the liver^79^ and visceral adipose tissue. A value close to the literature is found for the bulk heart^80^, without differentiating between muscle tissue and blood. The low SNR of the UTE in the lung region results in negative conductivity values^60^. We are not aware of any in vivo reference values for the other tissues listed in our study and we observe that the conductivity values encountered tend to be lower than the ex vivo values in these regions. All values should be treated with caution, e.g. considering the number of voxels or the ROI-wise standard deviation as a surrogate confidence metric as we suggested, and will benefit from comparative studies with more advanced methods from different sequences and manufacturers. Finally, with regard to the trends observed, we emphasize the increase in conductivity in all cerebral compartments with age which is at odds with published work on animal excised pieces^81^. In our working frequency range, the body is usually considered a as mixture of particles in the aqueous extracellular space which then reflects most of its EM behavior. Increased conductivity may then suggest an increase in ionic concentration, fluid content^42^ or even a change in the structure of the extracellular space over time.

While the biology of the aging brain is a complex process, some studies indicate an increase in brain water content, which is associated with neurodegenerative processes and cognitive decline^82,83^ while others suggest a decrease in an animal model^84^. These variations are closely linked^42,55^ to global conductivity in gray or white matter, for example, which would then be added to the list of parameters of interest for quantitative MRI applied to longitudinal studies. Furthermore, and this is one of the limitations of our study, we need to be able to say whether the evolution of EPs over time, and therefore of their physiological bases, is linear or not. Furthermore, the decrease in EEG power with age could be another indication of the overall increase in cerebral conductivity, even at low frequencies, with the addition of concomitant shunting effects^85^. The drop in conductivity in one of the two kidneys could also reflect these changes in free water concentration associated with the dehydration more frequently encountered in the elderly while the drop for aortic blood could be related to augmented whole blood viscosity^86^. The decreases in conductivity in several organs with respect to the fat fraction are consistent with the low theoretical conductivity of fat, whose increased level lowers simultaneously water content and ionic mobility. Finally, gender seems to be a good predictor of a difference in conductivity for the liver in particular. If the difference is real, it is once again probably associated with ionic substrates, such as iron content, or physico-chemical substrates, such as fat content^87^. Multi-variate regression provides initial clues to the potential independence of certain predictors (Table S1), such as age and FVF for liver. In contrast, there is a strong correlation between gender and age for the pancreas and aorta estimates, partly because older women are under-represented in our small cohort.

In conclusion, the main aim of our study was to establish a reliable protocol for estimating in vivo conductivity in several organs, compare our population values with reference ex vivo values and identify trends in relation to their characteristics. Our cohort is small and the results are preliminary but they suggest that in vivo conductivity values may deviate from the ex vivo reference values, and that inter-individual variations (e.g. vs. age or sex) may not be negligible. Beyond statistical power, the limitations of our study include the use of a simple reconstruction method and the low SNR/contrast in certain organs (such as lung or CSF) with the UTE sequence which could lead to estimation bias. As the variability of in vivo EPs has not been addressed extensively by existing studies, there is still no ground truth available for absolute quantification of conductivity in vivo, so our results will have to be confirmed by larger studies using different methods and MRI scanners. Should these correlations were to prove significant for larger cohorts, they might provide a new tool for studying structural and functional differences between individuals.

Overall, we believe that electromagnetic models for SAR management and patient safety, as well as the addition of electromagnetic parameters to clinical studies, will benefit greatly from these in vivo studies.

### Supplementary Information

Supplementary Figures.

## Data Availability

Data are available from the corresponding author upon reasonable request.
