# Supplementary material for: Changes of in vivo electrical conductivity in the brain and torso related to age, fat fraction and sex using MRI
Source: Sci Rep. 2024 Jul 12;14:16109. doi: 10.1038/s41598-024-67014-9 (PMC11245625; doi:10.1038/s41598-024-67014-9)
Supplement: Supplementary file 1 — Supplementary Figures. [file 41598_2024_67014_MOESM1_ESM.pdf]

Changes of in vivo electrical conductivity in the brain and torso related to age, fat fraction and sex using MRI

Zhongzheng He[1†], Paul Soullié[1†*], Pauline Lefebvre[1], Khalid Ambarki[2], Jacques Felblinger[1, 3], Freddy Odille[1, 3]

Figure S1. Dixon water images and reconstructed conductivity σ_{EPT} maps on two abnormal subjects: one with a benign liver tumor (Subject 1) and another with liver cysts (Subject 2). These images exhibit distinctive contrasts compared to normal tissue in both water and σ_{EPT} images.

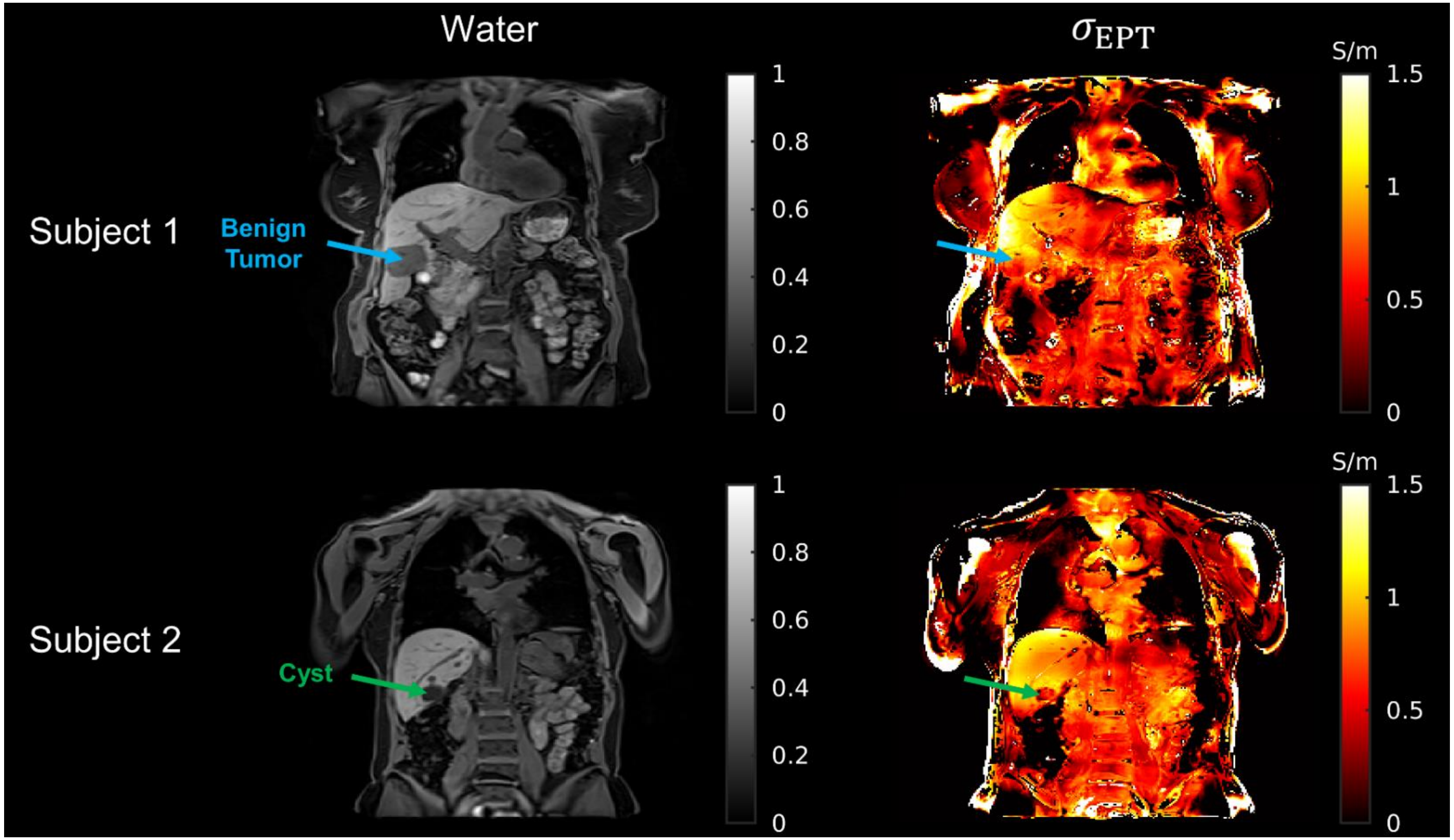

Table S1. Summary table for multivariate analyses. In each case, the two significant predictors are taken into account in the model and we display the associated p-values. To give an idea of the degree of independence between predictors, we also provide a simple estimate of correlation via linear regression. We find that there is a strong correlation between age and gender in our dataset, and we do indeed lack older women.

**ORGANS**	**V1 NAME**	**V2 NAME**	**MODEL1 $\sigma = 1 + V1 + V2$**		**MODEL2 V1=1+V2**
			**V1**	**V2**	**V2**
			p-value		
Right Kidney	Age	FVF	**0.014**	**0.027**	0.640
Liver	FVF	Sex	**0.004**	**<0.001**	0.603
Pancreas	Age	Sex	0.340	0.676	**0.003**
Duodenum	BMI	Sex	**0.031**	**<0.001**	0.954
Heart	FVF	Sex	**0.004**	**0.01**	0.322
Aorta	Age	Sex	0.079	0.051	**0.003**

Figure S2. Linear regression fitting plots illustrating the correlation between σ_{EPT} and age/FVF/BMI/Sex, with a focus on cases exhibiting a statistically significant p-value (< 0.05).

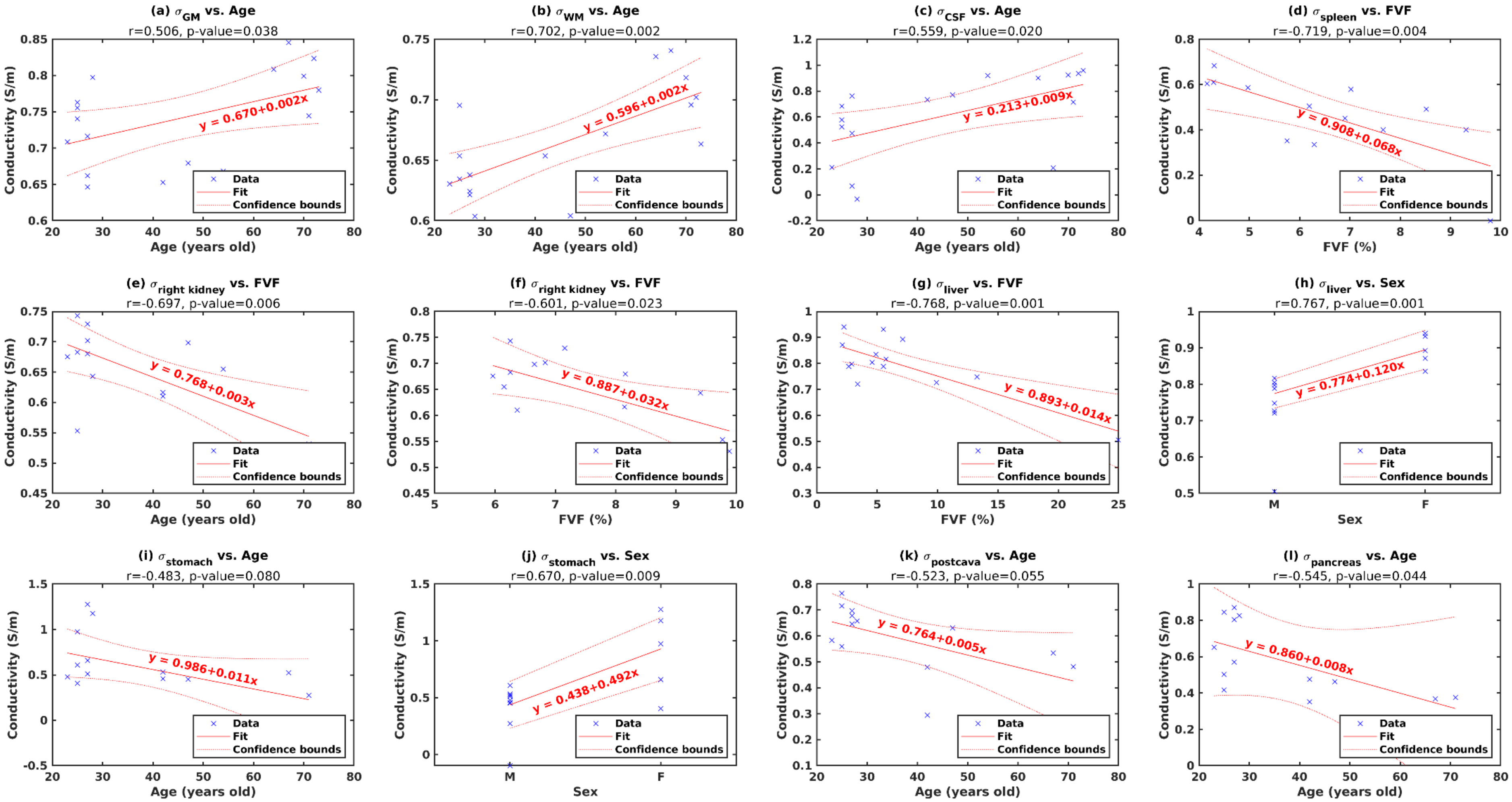

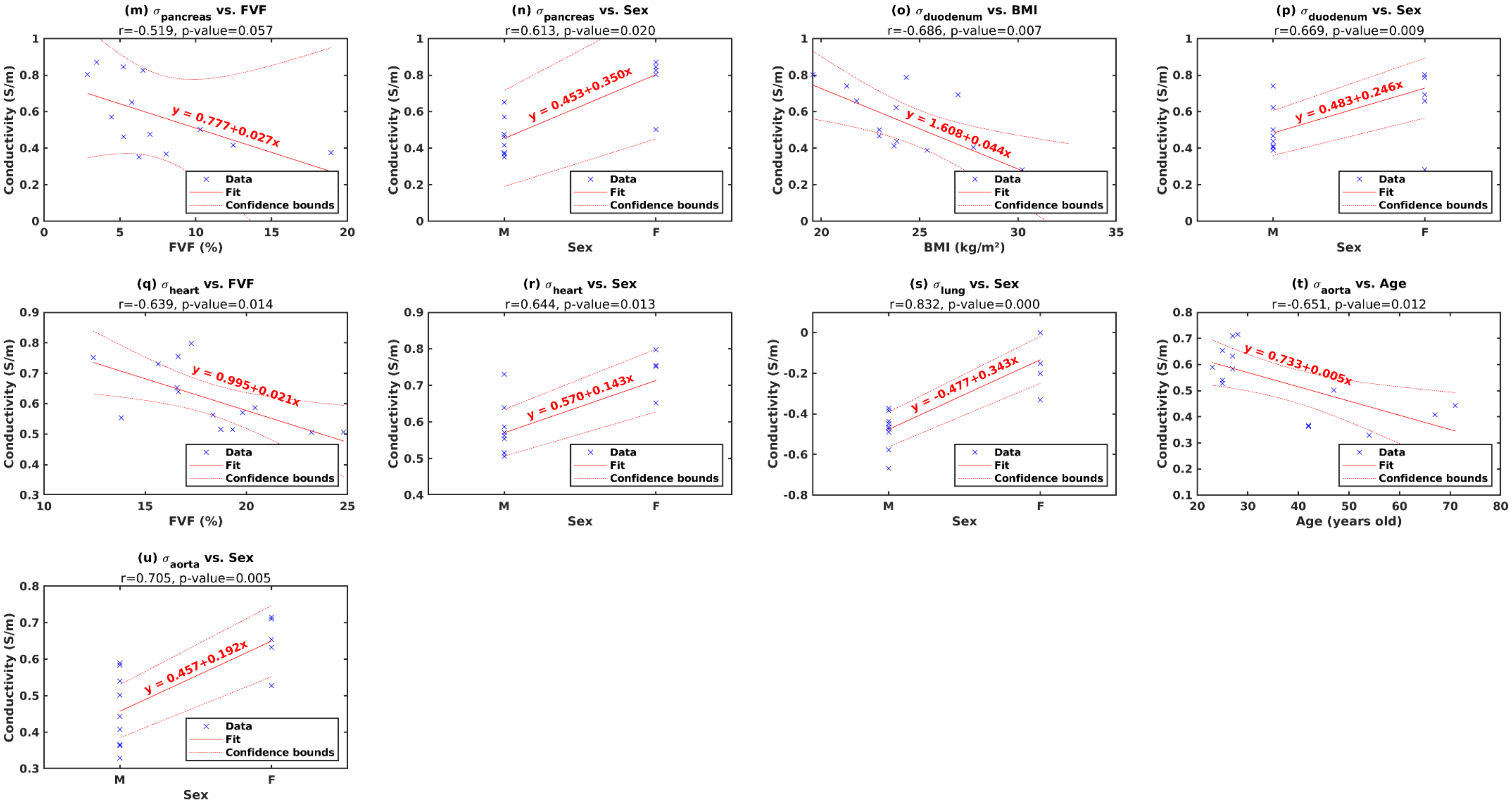

(m) σpancreas vs. FVF
r=-0.519, p-value=0.057
y = 0.777+0.027x
Conductivity (S/m)
FVF (%)
(n) σpancreas vs. Sex
r=0.613, p-value=0.020
y = 0.453+0.350x
Conductivity (S/m)
Sex
(o) σduodenum vs. BMI
r=-0.686, p-value=0.007
y = 1.608+0.044x
Conductivity (S/m)
BMI (kg/m²)
(p) σduodenum vs. Sex
r=0.669, p-value=0.009
y = 0.483+0.246x
Conductivity (S/m)
Sex
(q) σheart vs. FVF
r=-0.639, p-value=0.014
y = 0.995+0.021x
Conductivity (S/m)
FVF (%)
(r) σheart vs. Sex
r=0.644, p-value=0.013
y = 0.570+0.143x
Conductivity (S/m)
Sex
(s) σlung vs. Sex
r=0.832, p-value=0.000
y = -0.477+0.343x
Conductivity (S/m)
Sex
(t) σaorta vs. Age
r=-0.651, p-value=0.012
y = 0.733+0.005x
Conductivity (S/m)
Age (years old)
(u) σaorta vs. Sex
r=0.705, p-value=0.005
y = 0.457+0.192x
Conductivity (S/m)
Sex
Data
Fit
Confidence bounds
M
F
